# Detection of feline haemoplasma species in experimental infections by in-situ hybridisation

**DOI:** 10.1016/j.micpath.2010.09.003

**Published:** 2011-02

**Authors:** Iain R. Peters, Chris R. Helps, Barbara Willi, Regina Hofmann-Lehmann, Timothy J. Gruffydd-Jones, Michael J. Day, Séverine Tasker

**Affiliations:** a School of Veterinary Sciences, University of Bristol, Langford, Bristol, BS40 5DU, UK; b Clinical Laboratory, Vetsuisse Faculty, University of Zurich, Switzerland

**Keywords:** Haemoplasma, *Mycoplasma haemofelis*, “*Candidatus* Mycoplasma haemominutum”, “*Candidatus* Mycoplasma turicensis”, Fluorescence in-situ hybridisation

## Abstract

The aim of this study was to use fluorescence in-situ hybridisation (FISH) to search for the tissues and cell types important in survival and persistence of *Mycoplasma haemofelis*, “*Candidatus* Mycoplasma haemominutum” or “*Candidatus* Mycoplasma turicensis” in infected cats. A 16S rDNA probe for each species was applied to formalin-fixed, paraffin wax-embedded tissues sections collected from experimentally infected cats.

Tissues (*n* = 12) were collected, at necropsy, from ten cats which had been infected with *M. haemofelis*, and one each with “*Ca.* M. haemominutum” and “*Ca.* M. turicensis”. *M. haemofelis* specific hybridisation was present on red blood cells (RBCs) in all tissues from acutely infected cats, but not the majority of tissues from chronically infected cats. “*Ca.* M. haemominutum” specific hybridisation was present on scattered RBCs within the spleen and liver. Specific probe hybridisation was not detected in any of the “*Ca.* M. turicensis” infected tissues.

Haemoplasmas were detected on the surface of RBCs only and not any other cell type. Additionally, FISH was limited by sensitivity and could not detect the lower numbers of organisms present in tissues of cats chronically infected with *M. haemofelis*. Occasional organisms were detected in cats acutely infected with “*Ca.* M. haemominutum” but not “*Ca.* M. turicensis”.

## 1 Introduction

Feline infectious anaemia is caused by feline haemotropic mycoplasmas, also known as haemoplasmas, which are wall-less epicellular erythrocytic bacterial parasites which have not yet been cultured *in vitro*. Three distinct feline haemoplasmas species are recognised; *Mycoplasma haemofelis*, ‘*Candidatus* Mycoplasma haemominutum’ and ‘*Candidatus* Mycoplasma turicensis’ [6,10,11,20,22]. These three species differ in their pathogenicity. *M. haemofelis* infection often results in a severe haemolytic anaemia whilst “*Ca.* M. haemominutum” infection usually results in few clinical signs, although a drop in red blood cell (RBC) count can occur [1,5,14,18,19]. The third species, “*Ca.* M. turicensis”, originally reported from Switzerland, was associated with mild to severe anaemia in a naturally infected cat [20], however we have recently found it to result in negligible clinical signs in experimentally infected cats [9,18].

The development of sensitive and specific quantitative real-time polymerase chain reaction (qPCR) assays for detection of feline haemoplasma DNA [12–15,20] has allowed successful monitoring of the kinetics of haemoplasma copy number in the blood of experimentally infected cats. These experimental infections have shown a rapid increase in blood haemoplasma copy number in the post-infection period. However, apart from during this peak infection, organisms are infrequently visible on the surface of RBCs in blood smears [1,4,13–15,18,20]. Later in infection, marked variations in *M. haemofelis* copy number over time have been reported in Refs. [14,15] and these fluctuations can be as large as a 4 log difference over 2 or 3 days. One possible explanation for this rapid cycling during the chronic phase of infection is sequestration and subsequent release of organisms from organs such as the spleen, liver or lungs [8].

In-situ hybridisation (ISH) utilises a labelled nucleic acid probe to specifically identify the location of a pathogen within a host tissue, by hybridisation to a specific gene sequence and is particularly useful for unculturable organisms. ISH, with colourimetric detection, has been used to investigate acute but not chronic *M. haemofelis* infection in the liver and kidney of cats [2] and tissues from pigs infected with *Mycoplasma suis* [7]. These studies identified organisms associated with RBCs within the tissues but not with other cell types. The purpose of this study was to use fluorescence in-situ hybridisation (FISH) for the examination of tissues from cats infected experimentally with each of the feline haemoplasma species during the acute phase of infection to determine the cell types involved. Furthermore, tissues from *M. haemofelis* infected cats in the chronic phase of infection, including those thought to be undergoing copy number cycling, were used to determine cell types and organs that might be involved in *M. haemofelis* sequestration and copy number cycling.

## 2 Results

### 2.1 Optimisation of FISH methodology

None of the DIG-labelled probes bound to gDNA from a non-infected cat but all three were able to detect their respective 16S rDNA containing plasmid (minimum of ∼10^6^copies). The sensitivity of the probes for the non-target haemoplasma species was approximately 10-fold less than their target species. This cross-reactivity was not considered significant for these experiments as each cat was infected with only a single known haemoplasma species (confirmed by species-specific qPCR). All three probes detected the gDNA from the *M. haemofelis* infected blood, although the intensity of the signal was greatest with the *M. haemofelis* probe. The gDNA from cats infected with “*Ca.* M. haemominutum” and “*Ca.* M. turicensis” were of insufficient copy number to give a positive result by dot blot with either the target or non-target species probes.

**Fig. 1:**
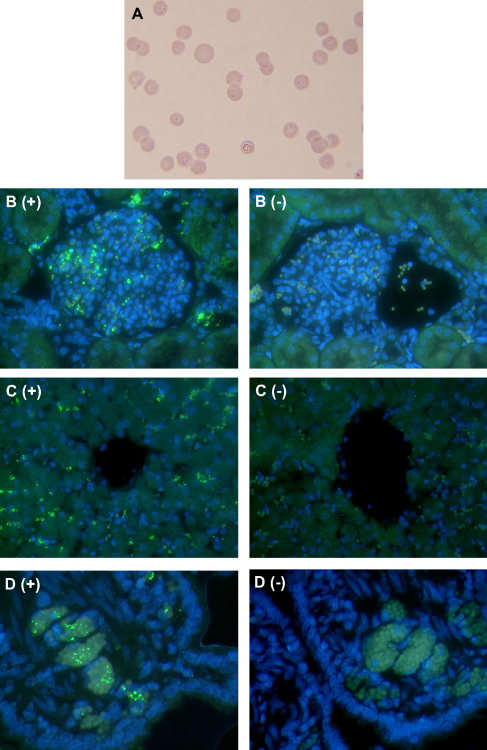
A Giemsa stained, EDTA-anticoagulated, peripheral blood smear (A) was made prior to collection of tissue sections from a *M. haemofelis* infected cat (HF11) 19 DPI when approximately 70% of RBCs had one or more organisms attached. Formalin-fixed, paraffin wax-embedded tissue sections were examined by FISH, using a DIG-labelled 16S rDNA probe, tyramide amplification and DTAF-labelled Streptavidin, with DAPI counterstain. *M. haemofelis* organisms (green) appear on the surface of RBCs within the glomeruli of the kidney (B+), sinusoids of the liver (C+), and blood vessels of other organs, including the jejunum (D+). The positive signal from *M. haemofelis* was not seen when a 100-fold excess of unlabelled probe was added to the hybridisation buffer (B−, C− and D−). (For interpretation of the references to colour in this figure legend, the reader is referred to the web version of this article).

Initial optimisation of the FISH protocol was attempted on blood smears obtained from cats HF11 and HF5 (highest blood 16S rDNA copy numbers) at the time of tissue collection (Table 1). The blood smears from these cats had been stained with Giemsa and approximately 70% of red blood cells were found to contain one or more *M. haemofelis* organism (Fig. 1A). Smears made at the same time were fixed in either methanol, acetone or 4% paraformaldehyde prior to performing FISH. Despite the presence of large numbers of organisms on the Giemsa-stained smears, no positive signal was detected on any of the blood smears tested by FISH.

The FISH procedure was also performed on tissues without the tyramide amplification system using appropriate fluorescent dye-conjugated reagents but no significant positive signals could be detected, therefore tyramide amplification was required for the FISH protocol.

### 2.2 FISH of tissue sections

Histopathological examination of the tissues collected from the “*Ca.* M. turicensis”, “*Ca.* M. haemominutum” and chronically infected *M. haemofelis* cats (Table 1) showed no significant pathology [17]. The tissues from the acutely anaemic cats (Table 1) had changes consistent with severe anaemia, including anoxia of centrilobular hepatocytes and bone marrow erythroid hyperplasia.

RBCs in the tissue sections showed significant autofluorescence and, when viewed with the broad pass filter, contrasted with the green from the DTAF (haemoplasma staining) and the blue from the DAPI counterstain (cell nuclei). This allowed easy localisation of the DIG probe in relation to RBCs. Haemoplasma-specific hybridisation was not seen in any of the negative control sections where the hybridisation buffer contained 100-fold excess of unlabelled probe or where the DIG-labelled probe was omitted. Probe hybridisation was detected in all tissues examined from the two cats with high *M. haemofelis* blood copy numbers (Table 1: HF5 and HF11). Specific signals were always co-localised with RBCs within all of the tissues examined and were associated with blood vessels, red pulp of the spleen, hepatic sinusoids, renal glomeruli and regions of the bone marrow sections with abundant autofluorescent RBCs (Fig. 1), but not to any other cell type. Within the bone marrow, there was no evidence of significant signals associated with RBC precursors since the signal-associated cells appeared to be mature erythrocytes.

Tissue sections from the cats with moderate *M. haemofelis* blood copy numbers (Table 1: HF9 and HF10) had only occasional positive signals on isolated RBCs within the liver and spleen (up to 1 per ×20 objective field). No positive signals were observed in any of the other tissue sections from the remaining *M. haemofelis* infected cats. With the exception of a few scattered RBCs within the spleen and liver of the “*Ca.* M. haemominutum” infected cat, no probe hybridisation was detected in the tissue sections from the “*Ca.* M. haemominutum” or “*Ca.* M. turicensis” infected cats.

The number of haemoplasma 16S rDNA copies was assessed by qPCR in the tissues collected from the “*Ca.* M. haemominutum” and “*Ca.* M. turicensis” infected cats, in order to confirm the presence of haemoplasma 16S rDNA in these tissues (Table 2). All tissues were positive, with the exception of the renal medulla from the “*Ca.* M. turicensis” infected cat.

## 3 Discussion

This study has documented the localisation of *M. haemofelis* organisms within the tissues of cats during the acute phase and chronic phases of *M. haemofelis* infection, and a cat with either acute “*Candidatus* Mycoplasma haemominutum” or “*Candidatus* Mycoplasma turicensis” infection. *M. haemofelis* organisms were localised to RBCs within the tonsil, submandibular salivary gland, bone marrow, lung, liver, spleen, kidney, jejunum, colon, mesenteric lymph node and colonic lymph node, but no evidence was found of localisation to other cell types. These results are similar to those of Berent et al., who using the same 16S rDNA probe and sections of liver and kidney, localised *M. haemofelis* organisms to RBCs only [2]. An ISH study of liver, lymph node, tonsil, spleen, heart, lung, kidney and small and large intestine collected from pigs infected with *M. suis* localised organisms to red blood cells within major blood vessels, hepatic sinusoids and renal glomeruli [7].

During the development of the FISH methodology, attempts were made to use peripheral blood smears prepared at the same time as tissue collection from the two cats with acute *M. haemofelis* infection (HF5, HF11). Despite using a number of fixatives and the subsequent success of the FISH procedure, no positive results were obtained. This may have been due to detachment and loss of organisms during the FISH procedure, destruction of 16S rDNA nucleic acid during fixation or failure of the probe to penetrate the organisms despite permeabilisation with proteinase K. Further optimisation of the FISH method on peripheral blood smears was not undertaken since the technique was applied successfully to the formalin-fixed tissues. Formalin fixation and routine processing of blood prior to embedding and sectioning may demonstrate whether this is a critical step for successful FISH and may be a consideration if this technique is applied to cryopreserved tissue sections.

Haemoplasma 16S rDNA has been detected in the saliva, faeces and urine of cats infected acutely with some of the haemoplasma species [3,9,21]. We found no evidence of localisation of organisms to any non-RBC cells within the kidney, intestine or salivary gland that may explain the presence of *M. haemofelis* DNA in saliva, faeces or urine. Therefore, these findings could be the result of transfer of infected RBCs into saliva, urine or faeces or translocation of organisms into saliva, urine or faeces from RBCs within blood vessels. Alternatively, organisms may be present associated with cell types other than RBC at levels below the detection limit of the FISH assay. A recent study has assessed the potential of saliva to transmit “*Ca.* M. turicensis” by oronasal and subcutaneous administration but none of the 13 cats became qPCR positive, although they did have evidence of seroconversion to haemoplasma-associated proteins [9]. It is therefore possible that the 16S rDNA present in saliva, urine or faeces is free within the sample due to lysis of the organisms or that the organisms have been rendered non-viable. Further work is required to determine whether intact organisms are present in these fluids and whether they are viable and thus a potential source for the spread of infection.

Positive FISH results were obtained from *M. haemofelis* infected cats only in the acute phase of infection (HF5 and HF11) with only a few scattered positive signals in the liver and spleen of two *M. haemofelis* infected cats in the chronic phase (HF9 and HF10). All positive signals were associated with RBCs and no other cell types were implicated in the replication or sequestration of these organisms. Haemoplasma 16S rDNA copy numbers have been quantified by qPCR in these tissues previously [17]. Tissues from the cats in the acute (HF5 and HF11) and non-cycling chronic phase (HF3, HF7, HF 9, HF10) (Table 1) were positive by qPCR, whereas those collected from the cats in the chronic phase of infection, where the collection coincided with a trough in blood 16S rDNA copy number (HF2, HF4, HF6, HF12), often had negative qPCR results. This suggests a limitation of FISH, which may require a relatively large number of organisms to be present for a positive result to be obtained. An approximate 10-fold reduction in haemoplasma blood copy number resulted in a dramatic reduction in the number of positive signals seen (e.g. results of HF5 and HF11 vs. HF 9 and HF10). FISH represents a more specific method of detection in comparison with stained blood smears, but still relies on the presence of sufficient organisms within the small amount of tissue on the microscope slide for detection. It is possible that detachment and loss of organisms during the FISH procedure may have accounted for the requirement of a high initial infective load for positive results. The ability of the probe to bind to its target and the method of signal detection will also affect the sensitivity, which was apparent in the dot blot studies as relatively large numbers of 16S rDNA plasmid gene copies were required (∼10^6^) for a positive result. Furthermore, the tyramide amplification system was required to detect the positive results as omission of this step resulted in no signal, which has been reported previously when colourimetric detection was used [2].

These factors affecting the sensitivity of detection may account for the low number of positive signals from the “*Ca.* M. haemominutum” infected tissues (HM3) as the tissue copy numbers were less than those reported previously for the two cats with acute *M. haemofelis* infection (HF5 and HF11) [17]. The copy numbers in the tissues from the cat with acute “*Ca.* M. turicensis” infection (TU3) also tended to be lower than that for the cats with *M. haemofelis* infection with the exception of the tonsil, mesenteric lymph node and the mid-jejunum, which had higher levels than both HF5 and HF11, as well as the colon and colonic lymph node that had a greater number of copies than the equivalent tissues from HF5 but not HF11. The lack of signal for the “*Ca.* M. turicensis” tissues with relatively greater copy numbers than the equivalent *M. haemofelis* tissues may be a result of variations in the composition of the sections examined versus those assayed by qPCR, particularly with regards to the number of RBCs present within them. Only one cat infected with “*Ca.* M. haemominutum” and one with “*Ca.* M. turicensis” was included in this study because the outcome of infection was considered predictable. In retrospect, more should have been infected; therefore the results for these two species should be interpreted with caution until a larger number of infected cats have been examined.

*M. haemofelis* infection has been associated with copy number cycling and this has been reported previously in four of the *M. haemofelis* infected cats (HF2, HF4, HF6, HF12) used in this study [17]. Sequestration and release of organisms from organs such as the liver, spleen or lungs has been suggested as a possible mechanism behind this phenomenon [8]. Analysis by qPCR of tissues from the cats used in this study did not identify any sites of sequestration, but did identify a greater number of *M. haemofelis* copies (than expected due to blood content alone) in the lung and spleen of cats with high or moderate blood copy numbers [17]. No evidence of localisation of the *M. haemofelis* organisms to a cell type other than RBCs was found in any sections of lung or spleen to explain the higher than expected copy numbers and no organisms were observed in these tissues from animals undergoing copy number cycling. It is possible that vascular congestion occurring *post-mortem* or the high level of perfusion of these organs *intra vitam* could have contributed to the higher levels of *M. haemofelis* DNA detected by qPCR in the tissues from these cats.

This study has demonstrated that during the acute phase of infection with *M. haemofelis*, organisms are closely associated with RBCs within a wide range of tissues. In addition, no other cell types were identified with haemoplasmas present during the acute phase or as important reservoirs for survival, persistence, or sequestration during the chronic phase, including during copy number cycling. Furthermore, the study has demonstrated the limitations of the sensitivity of FISH for the investigation of haemoplasma infections, and the possibility of sites of persistence or sequestration cannot be ruled out from these results due to this lack of sensitivity.

## 4 Materials and methods

### 4.1 Blood and tissue samples

Twelve barrier-maintained, specific pathogen free (SPF) derived, domestic-shorthaired cats, aged 7 months with six neutered males and six entire males, were used in the study. Cats were infected experimentally with either *M. haemofelis* (*n* = 10; Cats HF2-7 and HF9-12), “*Ca.* M. haemominutum” (*n* = 1; HM3) or “*Ca.* M. turicensis” (*n* = 1; TU3). Details of the experimental procedures, infective doses administered and infection monitoring procedures have been described previously [17,18]. The discontinuous numbering of the cats was due to the inclusion of additional cats for a parallel study documenting haematological changes, Coombs’ test positivity and blood glucose concentrations in haemoplasma-infected cats [18]. All procedures and experiments described were undertaken under a project license approved under the UK Animals (Scientific Procedures) Act 1986.

Blood samples (EDTA-anticoagulated) were collected by jugular venipuncture for haemoplasma real-time PCR (qPCR) [12] on day 0 of the study in order to ensure that the animals had no pre-existing infection, and regularly thereafter (minimum of three times weekly) to monitor progress of the infection [18]. Blood smears were prepared immediately after mixing with EDTA and were rapidly air dried and either stained with Giemsa or used for optimisation of the FISH protocol (on organosilane-coated slides) following fixation with methanol, acetone or 4% paraformaldehyde.

Tissues were collected, during *post-mortem* examination, at various times post-infection to determine the localisation of haemoplasma organisms during different phases of the infection. For the basis of this study, cats were classified as acutely infected if they were sampled during the initial phase of infection during which there is a rise in haemoplasma copy number (<22 days post-infection [DPI]) and when, in the case of *M. haemofelis* infection, the cats were anaemic (PCV <25%) (Table 1). Cats were considered to be in the chronic phase of infection if their haemoplasma copy number had reduced from its peak and was either relatively stable, or had decreased to the point that some negative qPCR results were obtained (<10 copies per 5 μl of blood; 98–224 DPI). *M. haemofelis* infected cats were not anaemic at this point. For the cats with chronic *M. haemofelis* infection, tissues were collected at times when copy number cycling was believed to be occurring (Table 1) [17]. Tissues collected at necropsy examination comprised tonsil, submandibular salivary gland, bone marrow, lung, liver, spleen, kidney (cortex and medulla), jejunum, colon, mesenteric lymph node and colonic lymph node. Tissues were fixed in 10% neutral-buffered formalin and embedded in paraffin wax using standard techniques. A set of sections stained by haematoxylin and eosin (HE) was prepared from these tissues and these were subject to histopathological examination by a board-certified veterinary pathologist (MJD). The number of haemoplasma 16S rDNA copies was assessed by qPCR in the tissues collected from the “*Ca.* M. haemominutum” and “*Ca.* M. turicensis” infected cats, in order to confirm the presence of haemoplasma 16S rDNA in these tissues using the methods described previously [17].

### 4.2 Probe synthesis

The *M. haemofelis* 16S rDNA probe was identical to that described previously [2]. A 393 base pair (bp) probe was synthesised using a modified (underlined nucleotides) forward primer (5′-GATCTTGGTTTCGGCCAAGG-3′) and the original reverse primer (5′-CGAAGTACTATCATAATTATCCCTC-3′) [1] (Invitrogen Ltd., Paisley, Scotland) using HotStar Taq DNA Polymerase (Qiagen, Crawley, UK) and PCR digoxigenin (DIG) Probe Synthesis Mix (10×) from the PCR DIG Probe Synthesis Kit (Roche Diagnostics Ltd., Burgess Hill, West Sussex). Each 100 μl synthesis reaction contained 10 μl of 10× Qiagen PCR buffer, 5units of HotStar Taq Polymerase, 3 mM MgCl_2_, 200 nM of forward and reverse primer, 10 μl of 10× PCR DIG Probe Synthesis mix (200 μM dATP, dCTP and dGTP, 130 μM dTTP, and 70 μM DIG-dUTP: final concentration) and 10^6^ copies of a plasmid containing the *M. haemofelis* 16S rDNA gene (GenBank accession no. genbank:%3Ca href = AY150985) [16]. The forward primer sequence was altered by transposing the underlined bases in order to match the sequence in the plasmid used for probe synthesis and 68% of *M. haemofelis* sequences available in GenBank (accessed on 22nd February 2010). Reactions were heated to 95 °C for 15 min and then 40 cycles of 95 °C for 30 s, 55 °C for 30 s and 72 °C for 30 s before final incubation at 72 °C for 7 min in a PTC-200 DNA engine (Bio-Rad Laboratories, Hemel Hempstead, UK). The probe was purified from the PCR mix using the Macherey–Nagel NucleoSpin Extract II kit (ABgene, Epsom, UK) and the concentration measured using the Qubit Quatitation Platform (Invitrogen) before being stored at −20 °C prior to use. An unlabelled PCR product was also synthesised for use as a negative control.

Probes for similar regions of the 16S rDNA gene from “*Ca.* M. haemominutum” and “*Ca.* M. turicensis” were synthesised using primers (forward: GAACGGGYGAGTAAYACATA, reverse: 5′CACATAGTTWGCTGTCACTTATTCA-3′) designed with Primer3 (http://frodo.wi.mit.edu/primer3/), which amplified 421 (GenBank accession no. genbank:%3Ca href = AY150981) and 393bp (GenBank accession no. genbank:%3Ca href = AY831867) products, respectively, from species-specific 16S rDNA plasmids [16,20], using reaction mixes and thermocycling protocols as described above.

The specificity of the probes was evaluated against dilutions of the species-specific plasmids (10^8^, 10^7^, 10^6^, 10^5^ copies per spot) and genomic (g) DNA isolated from feline blood obtained from *M. haemofelis* (∼10^7^ copies per spot), “*Ca.* M. haemominutum” (∼10^6^ copies per spot) and “*Ca.* M. turicensis” (∼10^5^ copies per spot) infected cats and a non-infected SPF cat. Plasmid dilutions and gDNA were heated to 100 °C and quenched on ice before spotting onto nylon membrane (Hybond-N+, GE Healthcare Life Sciences, Little Chalfont, Buckinghamshire), allowed to air dry, and UV cross-linked before pre-hybridisation for 1 h at 42 °C in hybridisation buffer (50% (v/v) formamide, 5× SSC, 0.02% (w/v) non-fat milk, 5% (w/v) dextran sulphate [20× SSC = 3 M NaCl, 0.3 M sodium citrate]). Membranes were then incubated overnight at 42 °C in hybridisation buffer containing 100 ng/ml of DIG-labelled probe that had been heated to 100 °C and quenched on ice before addition. Following hybridisation, blots were washed twice with 2× SSC at 60 °C for 10 min, twice with 0.2× SSC at 42 °C for 10 min and once with 0.1× SCC at room temperature for 5 min. Hybridisation was detected using the DIG Wash and Block Buffer Set, anti-digoxigenin-alkaline phosphatase conjugate, Fab’ fragment and CDP-star (all from Roche Diagnostics) as per manufacturer’s instructions before exposure to Kodak BioMax film (Sigma–Aldrich Company Ltd., Poole, Dorest).

### 4.3 Fluorescence in-situ hybridisation

Sections (0.6 μm) were cut from paraffin wax-embedded tissues onto organosilane-coated microscope slides, heated to 60 °C for 5 min to attach the sections, and dewaxed in histoclear (Fisher Scientific Ltd., Leicestershire, UK) for 10 min. Sections were rehydrated through an alcohol gradient (100% and 70% ethanol) for 10 min each before washing in DEPEC water for 2 min and DEPEC phosphate buffered saline (PBS; 0.15 M, pH 7.4) for 10 min. Endogenous peroxidase was blocked by incubation with 0.3% H_2_O_2_ in DEPEC PBS for 30 min before washing three times in PBS for 5 min each. Sections were permeabilised with 5 μg/ml proteinase K (Sigma–Aldrich) in 20 mM Tris pH 7.4, 0.1% (w/v) CaCl_2_ for 10 min before the reaction was stopped by incubation in 0.1 M glycine in DEPEC PBS for 5 min. Slides were washed three times for 5 min in PBS and then dehydrated through graded alcohols (70% and 100% ethanol). A gene frame (ABgene) was applied to the periphery of the section before being overlaid with 125 μl of hybridisation solution (50% [v/v] formamide, 2× SSC, 0.02% [w/v] non-fat milk, 10% [w/v] dextran sulphate and 200 ng/ml of DIG-labelled probe). Negative control slides were incubated with either hybridisation solution containing 100-fold excess of unlabelled probe or by omitting the DIG-labelled probe. Hybridisation was performed in a PTC-200 DNA engine with Twin Tower block (Bio-Rad Laboratories Ltd.) and slides were heated to 95 °C for 10 min and then hybridised overnight at 37 °C. Following hybridisation, slides were rinsed in an Omni Slide Wash Module (Thermo Fisher Scientific Inc, Loughborough, Leicestershire) twice with 2× SSC at 60 °C for 10 min, twice with 0.2× SSC at 42 °C for 10 min and once with 0.1× SSC at room temperature for 5 min.

Signal amplification was carried out with the Tyramide Signal Amplification (TSA) Biotin System (PerkinElmer, Waltham, Massachusetts). Sections were edged using an ImmEdge pen (Vector Labs, Peterborough, UK) and overlaid with TNB buffer (from TSA kit) with 2% (v/v) goat serum (Sigma–Aldrich) for 1hr at room temperature. All incubations were carried out in a humidified chamber in the dark. Sections were then incubated overnight at 5 °C with 1:100 sheep anti-digoxigenin-POD, Fab’ fragments in TNB buffer with 5% (v/v) goat serum. Slides were washed three times in PBS + 0.05% (v/v) Tween 20 (PBST), incubated with 1:50 tyramide solution for 10 min, washed three times in PBS, incubated with 1:1000 streptavidin-DTAF (Stratech, Newmarket, Suffolk) at room temperature for 1hr and washed three times in PBST. Sections were mounted with Vectashield HardSet Mounting Medium with DAPI (Vector Labs) and viewed using a Leica DMRB microscope (Leica UK, Milton Keynes, UK) with the Blue/Green/Red filter cube. Images were captured using a Colour Coolview charge coupled device camera (Photonic Sciences, Robertsbridge, East Sussex) and Image-Pro Plus software (Media Cybernetics, Baltimore, MD).

## Figures and Tables

**Table 1:** Details of animals used for tissue collection.

	HF5	HF11	HM3	TU3	HF3	HF7	HF9	HF10	HF2	HF12	HF4	HF6
Sex	MN	MN	F	F	F	F	F	F	MN	MN	MN	MN
DPI	13	19	22	20	98	98	104	111	149	167	177	224
Copy Number (per 5 μl blood)	13,000,000	65,000,000	2,300,000	1300	590,000	360,000	1,400,000	1,300,000	6.7^a^	0.76^a^	0.27^a^	2.3
Relative Level	High	High	Moderate	Low	Moderate	Moderate	Moderate	Moderate	Very Low	Very Low	Very Low	Very Low
Phase of Infection	Acute anaemic	Acute anaemic	Acute non-anaemic	Acute non-anaemic	Chronic no cycling	Chronic no cycling	Chronic no cycling	Chronic no cycling	Chronic cycling	Chronic cycling	Chronic cycling	Chronic cycling

This table shows the sex, number of days post-infection (DPI) when the tissues were collected as well as the number of haemoplasma 16S rDNA gene copies present in 5 μl of blood. HF cats were infected with *M. haemofelis*, the HM cat with “*Ca.* M. haemominutum” and the TU cat with “*Ca.* M. turicensis”. The relative level of 16S rDNA copies is indicated as high, moderate, low or very low. The phase of infection (acute or chronic), whether the animal was anaemic and whether copy number cycling was occurring in the chronic phase before tissue collection is also indicated.

a Average *M. haemofelis* blood copy number was calculated by repeated haemoplasma qPCR analysis, performed eight times, using replicate aliquots of the same extracted DNA preparations to assess how repeatedly low or negative the copy numbers results were.

**Table 2:** Number of haemoplasma 16S rDNA copies in 0.5 mg of tissue from HM3 and TU2.

Tissue	HM3	TU3
Tonsil	407	34,934
Colon	188	9544
Submandibular salivary gland	539	6900
Mesenteric lymph node	1085	86,642
Lung	10,216	269
Colonic lymph node	124	2011
Liver	2699	3606
Bone marrow	379	8382
Spleen	20,595	86,642
Kidney Cortex	2039	21
Kidney Medulla	202	Negative
Mid-jejunum	379	105,259

This table shows the haemoplasma 16S rDNA copy number per 0.5 mg tissue, determined by qPCR, in the samples collected from the “*Ca.* M. haemominutum” (HM3) and “*Ca.* M. turicensis” (TU3) infected cats. These results are calculated in the same way as those reported previously for the tissues from the *M. haemofelis* infected cats [17].

## References

[1] Berent L.M., Messick J.B., Cooper S.K. (1998). Detection of *Haemobartonella felis* in cats with experimentally induced acute and chronic infections, using a polymerase chain reaction assay. Am J Vet Res.

[2] Berent L.M., Messick J.B., Cooper S.K., Cusick P.K. (2000). Specific in situ hybridization of *Haemobartonella felis* with a DNA probe and tyramide signal amplification. Vet Pathol.

[3] Dean R.S., Helps C.R., Gruffydd Jones T.J., Tasker S. (2008). Use of real-time PCR to detect *Mycoplasma haemofelis* and ‘*Candidatus* Mycoplasma haemominutum’ in the saliva and salivary glands of haemoplasma-infected cats. J Feline Med Surg.

[4] Dowers K.L., Olver C., Radecki S.V., Lappin M.R. (2002). Use of enrofloxacin for treatment of large-form *Haemobartonella felis* in experimentally infected cats. J Am Vet Med Assoc.

[5] Foley J.E., Harrus S., Poland A., Chomel B., Pedersen N.C. (1998). Molecular, clinical, and pathologic comparison of two distinct strains of *Haemobartonella felis* in domestic cats. Am J Vet Res.

[6] Foley J.E., Pedersen N.C. (2001). ‘*Candidatus* Mycoplasma haemominutum’, a low-virulence epierythrocytic parasite of cats. Int J Syst Evol Microbiol.

[7] Ha S.K., Jung K., Choi C., Ha Y., Song H.C., Lim J.H. (2005). Development of in-situ hybridization for the detection of *Mycoplasma haemosuis* (Eperythrozoon suis) in formalin-fixed, paraffin wax-embedded tissues from experimentally infected splenectomized pigs. J Comp Pathol.

[8] Maede Y. (1978). Studies on feline haemobartonellosis. V. Role of the spleen in cats infected with Haemobartonella felis. Jpn J Vet Sci.

[9] Museux K., Boretti F.S., Willi B., Riond B., Hoelzle K., Hoelzle L.E. (2009). In vivo transmission studies of ’ *Candidatus* Mycoplasma turicensis’ in the domestic cat. Vet Res.

[10] Neimark H., Johansson K.E., Rikihisa Y., Tully J.G. (2001). Proposal to transfer some members of the genera Haemobartonella and Eperythrozoon to the genus mycoplasma with descriptions of ‘*Candidatus* mycoplasma haemofelis’, ‘*Candidatus mycoplasma* haemomuris’, ‘*Candidatus* mycoplasma haemosuis’ and ‘*Candidatus* mycoplasma wenyonii’. Int J Syst Evol Microbiol.

[11] Neimark H., Johansson K.E., Rikihisa Y., Tully J.G. (2002). Revision of haemotrophic Mycoplasma species names. Int J Syst Evol Microbiol.

[12] Peters I.R., Helps C.R., Willi B., Hofmann-Lehmann R., Tasker S. (2008). The prevalence of three species of feline haemoplasmas in samples submitted to a diagnostics service as determined by three novel real-time duplex PCR assays. Vet Microbiol.

[13] Tasker S., Caney S.M., Day M.J., Dean R.S., Helps C.R., Knowles T.G. (2006). Effect of chronic feline immunodeficiency infection, and efficacy of marbofloxacin treatment, on ‘*Candidatus* Mycoplasma haemominutum’ infection. Microbes Infect.

[14] Tasker S., Caney S.M., Day M.J., Dean R.S., Helps C.R., Knowles T.G. (2006). Effect of chronic FIV infection, and efficacy of marbofloxacin treatment, on *Mycoplasma haemofelis* infection. Vet Microbiol.

[15] Tasker S., Helps C.R., Day M.J., Gruffydd-Jones T.J., Harbour D.A. (2003). Use of real-time PCR to detect and quantify *Mycoplasma haemofelis* and “*Candidatus* Mycoplasma haemominutum” DNA. J Clin Microbiol.

[16] Tasker S., Helps C.R., Day M.J., Harbour D.A., Shaw S.E., Harrus S. (2003). Phylogenetic analysis of hemoplasma species: an international study. J Clin Microbiol.

[17] Tasker S., Peters I.R., Day M.J., Willi B., Hofmann-Lehmann R., Gruffydd-Jones T.J. (2009). Distribution of Mycoplasma haemofelis in blood and tissues following experimental infection. Microb Pathog.

[18] Tasker S., Peters I.R., Papasouliotis K., Cue S.M., Willi B., Hofmann-Lehmann R. (2009). Description of outcomes of experimental infection with feline haemoplasmas: copy numbers, haematology, Coombs’ testing and blood glucose concentrations. Vet Microbiol.

[19] Westfall D.S., Jensen W.A., Reagan W.J., Radecki S.V., Lappin M.R. (2001). Inoculation of two genotypes of *Hemobartonella felis* (California and Ohio variants) to induce infection in cats and the response to treatment with azithromycin. Am J Vet Res.

[20] Willi B., Boretti F.S., Cattori V., Tasker S., Meli M.L., Reusch C. (2005). Identification, molecular characterization, and experimental transmission of a new hemoplasma isolate from a cat with hemolytic anemia in Switzerland. J Clin Microbiol.

[21] Willi B., Boretti F.S., Meli M.L., Bernasconi M.V., Casati S., Hegglin D. (2007). Real-time PCR investigation of potential vectors, reservoirs, and shedding patterns of feline hemotropic mycoplasmas. Appl Environ Microbiol.

[22] Willi B., Tasker S., Boretti F.S., Doherr M.G., Cattori V., Meli M.L. (2006). Phylogenetic Analysis of “*Candidatus* Mycoplasma turicensis” isolates from pet cats in the United Kingdom, Australia, and South Africa, with analysis of risk factors for infection. J Clin Microbiol.

